# Common miRNA Patterns of Alzheimer’s Disease and Parkinson’s Disease and Their Putative Impact on Commensal Gut Microbiota

**DOI:** 10.3389/fnins.2019.00113

**Published:** 2019-03-05

**Authors:** Charlotte Hewel, Julia Kaiser, Anna Wierczeiko, Jan Linke, Christoph Reinhardt, Kristina Endres, Susanne Gerber

**Affiliations:** ^1^Faculty of Biology, Institute for Developmental Biology and Neurobiology, Center of Computational Sciences Mainz (CSM), Johannes Gutenberg University Mainz, Mainz, Germany; ^2^Center for Thrombosis and Hemostasis (CTH), University Medical Center of the Johannes Gutenberg University Mainz, Mainz, Germany; ^3^Department of Psychiatry and Psychotherapy, University Medical Center of the Johannes Gutenberg University Mainz, Mainz, Germany

**Keywords:** micro-RNAs, gut microbiome, crosstalk, neurodegeneration, Alzheimer’s disease, Parkinson’s disease

## Abstract

With the rise of Next-Generation-Sequencing (NGS) methods, Micro-RNAs (miRNAs) have achieved an important position in the research landscape and have been found to present valuable diagnostic tools in various diseases such as multiple sclerosis or lung cancer. There is also emerging evidence that miRNAs play an important role in the pathogenesis of neurodegenerative diseases such as Alzheimer’s disease (AD) or Parkinson’s disease (PD). Apparently, these diseases come along with changes in miRNA expression patterns which led to attempts from researchers to use these small RNA species from several body fluids for a better diagnosis and in order to observe disease progression. Additionally, it became evident that microbial commensals might play an important role for pathology development and were shown to have a significantly different composition in patients suffering from neurodegeneration compared with healthy controls. As it could recently be shown that secreted miRNAs are able to enter microbial organisms, it is conceivable that the host’s miRNA might affect the gut microbial ecosystem. As such, miRNAs may inherit a central role in shaping the “diseased microbiome” and thereby mutually act on the characteristics of these neurodegenerative diseases. We have therefore (1) compiled a list of miRNAs known to be associated with AD and/or PD, (2) performed an *in silico* target screen for binding sites of these miRNA on human gut metagenome sequences and (3) evaluated the hit list for interesting matches potentially relevant to the etiology of AD and or PD. The examination of protein identifiers connected to bacterial secretion system, lipopolysaccharide biosynthesis and biofilm formation revealed an overlap of 37 bacterial proteins that were targeted by human miRNAs. The identified links of miRNAs to the biological processes of bacteria connected to AD and PD have yet to be validated via *in vivo* experiments. However, our results show a promising new approach for understanding aspects of these neurodegenerative diseases in light of the regulation of the microbiome.

## Introduction

Micro RNAs (miRNAs) are an important subclass of small regulatory RNA. They are present in virtually all kingdoms of life and involved in crucial metabolic functions. Their main function is the downregulation of a target transcript via complementary base pairing. In general, these transcriptional modifiers are located within the synthesizing cell but are also able to translocate via gap junctional cell-cell contacts or exosomal release (Lemcke et al., 2015; Zong et al., 2016). They influence a variety of cellular processes such as autophagy, differentiation and stress-response (Zhu et al., 2009; Leung and Sharp, 2010; Mendell and Olson, 2012; Vidigal and Ventura, 2015; Gozuacik et al., 2017). Moreover, miRNAs have been found to contribute to pathological conditions, for example cancer development or autoimmunity (Xiao and Rajewsky, 2009). In the case of neurodegenerative diseases such as Parkinson’s disease (PD) or Alzheimer’s disease (AD), miRNA patterns of patients differ from healthy control groups. Recent examples for differential miRNA abundances are selective upregulations of let-7b and let-7e in cerebrospinal fluid (CSF) within AD patients (Derkow et al., 2018), or miR-24 and miR-205 that distinguished PD patients from controls in a comparably small cohort (Marques et al., 2017).

Many characteristics are shared between AD and PD, such as pathological mechanisms (e.g., protein aggregation) or genetic variants. Therefore, it has been postulated that these diseases may share similar alterations that could trigger comparable pathological pathways. Using literature mining of GWAS studies, regulatory networks enriched with AD- and PD-associated genes could be identified (Ramanan and Saykin, 2013; Sys et al., 2018). Nowadays miRNAs can be extracted from nearly every kind of body fluid or type of tissue (Weber et al., 2010), providing easy access to this information. Interestingly, it was already demonstrated in 2009 that miRNAs from feces might serve as biomarkers for colon cancer and active ulcerative colitis (Ahmed et al., 2009). This has been confirmed by many subsequent studies (Link et al., 2010; Kuo, 2013; Ren et al., 2015; Yau et al., 2016).

miRNAs within feces usually originate from exfoliated cells of the host’s gastrointestinal tract, but also possibly from food (Koga et al., 2010). Evidence for this was acquired by feeding mice with RNA obtained from *Brassica oleracea*. Exogenous plant miRNA was detectable within the animal’s serum, feces and tissues for up to 72 h (Liang et al., 2014). Interestingly, not only epithelial cells in the walls of the gut lumen are affected by the excreted or ingested miRNAs, but also the host’s microbiome. Liu and colleagues reported in 2016 that host miRNA can enter bacterial cells and regulate growth behavior and certain bacterial gene transcripts (Qin and Wade, 2018). In their study, *Fusobacterium nucleatum* cultured with human miR-515-5p showed an increased ratio of 16S rRNA/23S rRNA transcripts.

A different approach showed that it is possible to change the microbiome through miRNAs *in vivo*. Du et al. (2018) infected miR-146a-deficient mice with *Listeria monocytogenes*. This led to a differently developed gut microbiome and an increased infection tolerance against *Listeria monocytogenes* compared to wild type mice.

With respect to neurodegenerative diseases, the connection between host and gut microbiome has shifted increasingly into the focus of research (Scheperjans et al., 2015; Brandscheid et al., 2017; Cattaneo et al., 2017; Vogt et al., 2017; Bäuerl et al., 2018; Brenner et al., 2018; Gerhardt and Mohajeri, 2018). The contribution of altered miRNA patterns toward these changes and their possible impact on pathology is still an open question. Until now, miRNA analyses of feces collected from patients suffering from neurodegenerative diseases are rather scarce. However, consistent changes of miRNAs (miR-16, miR-21, mir-34a and miR-222) have been detected in both plasma and feces derived from the same individual (Tarallo et al., 2014).

This encouraged us to conduct the following two-step analysis. The first step was the collection of common miRNA patterns within blood and tissue from AD and PD patients, based on the data from seven studies. The second step consisted in identifying potential bacterial target sites for the selected miRNAs.

## Materials and Methods

### Differentially Expressed-miRNAs

Lists of differentially expressed miRNAs in AD and PD were aggregated from seven different studies (see Supplementary Table S1, Martins et al., 2011; Soreq et al., 2013; Burgos et al., 2014; Gui et al., 2015; Love et al., 2015; Lugli et al., 2015; Ding et al., 2016; Santoro et al., 2018). The lists were filtered after a *p*-value cut-off of 5%; the respective micro-RNAs were split in AD, PD up- and downregulated. The resulting lists were used as an input for the target scan analysis.

### Target Scan Analysis

Target Scan analysis for potential targets of the micro-RNAs on the human gut metagenome was performed with miRanda (v3.3a; Enright et al., 2003). Parameters for target filtering were: -strict -go -2 -ge -8 -sc 150.00 -en -25.00 -scale 3.00. For the target scan, the gut metagenome database was created based on the work by Qin et al. (2010). However, we only used species with a complete annotation available via the NCBI RefSeq (O’Leary et al., 2016). Afterwards, the miRanda hits were grouped by micro-RNA family affiliation as well as by organism and aggregated on family level. Afterwards, the miRanda output was parsed using a custom bash script. We intersected the miRanda output with the annotation file for each respective species and kept all protein IDs that were targeted (Supplementary Table S5). The protein IDs for the hits were used for the subsequent target analysis.

### GO-Terms for Human miRNA Targets on Bacterial Genes

GO terms were retrieved via GORetriever (McCarthy et al., 2006). The dataset was reduced using the GOSlimViewer with the metagenomic GOSlim set. Enrichment was done with Blast2GO (Gotz et al., 2008).

## Results

### miRNAs Differentially Expressed in AD and PD

**FIGURE 1:**
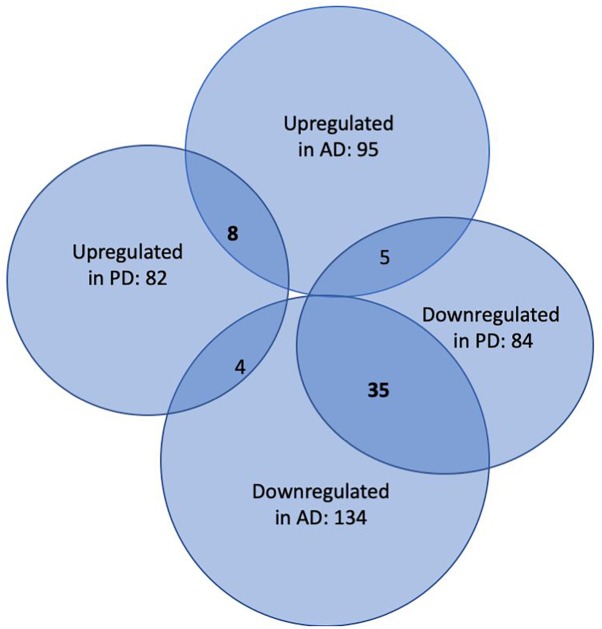
Venn-Diagram of miRNAs being differentially expressed in patients suffering from AD and PD versus healthy controls. The overlaps summarize miRNAs being detected in both conditions.

In the first step of our meta-analysis, 346 miRNAs were aggregated from 7 studies evaluating miRNAs in AD and PD (see Supplementary Tables S1, S8). The miRNAs were grouped into different categories, according to their up- or downregulation and their role in AD or PD. We were particularly interested in miRNAs being differentially expressed in both diseases. Figure 1 summarizes the number of miRNAs being up- or downregulated in both, AD and PD patients, compared to healthy controls (*p*-value < 0.5). Out of 95 miRNAs upregulated in AD patients, 8 miRNAs were upregulated in patients with PD as well (see also Table 1). The total number of upregulated miRNAs in PD compared to controls is 82. The comparison of downregulated miRNAs in both, AD patients (134 miRNAs) and PD patients (84 miRNAs), reveals a number of 35 expressed miRNAs (see also Table 2). Interestingly, 5 miRNAs found to be upregulated in AD were significantly downregulated in PD. Vice versa, 4 miRNAs that were found to be downregulated in AD were upregulated in PD. All candidates are listed in the Supplementary Table S2.

**Table 1:** Commonly upregulated miRNAs in AD and PD.

Identifier	Pathway Analysis Results	AD reference	PD reference
hsa-miR-30c-2-3p		Burgos et al., 2014	Burgos et al., 2014
hsa-miR-592		Burgos et al., 2014	Burgos et al., 2014
hsa-miR-148a-3p		Burgos et al., 2014	Burgos et al., 2014
hsa-miR-30a-5p		Burgos et al., 2014	Burgos et al., 2014
hsa-miR-190a-5p		Burgos et al., 2014	Burgos et al., 2014
hsa-miR-769-3p		Burgos et al., 2014	Tatura et al., 2016
hsa-mir-378c^∗^	Biofilm formation (*P. aeruginosa*), Bacterial Secretion Pathway, Quorum Sensing	Burgos et al., 2014	Soreq et al., 2013
hsa-mir-485-5p		Gui et al., 2015	Gui et al., 2015


^∗^*miRNAs that are involved in one of the analyzed pathways (see Results: Pathway analysis)*.

**Table 2:** Commonly downregulated miRNAs in AD and PD.

Identifier	Pathway Analysis Results	AD reference	PD reference
hsa-miR-124-3p		Burgos et al., 2014	Burgos et al., 2014
hsa-miR-127-3p		Burgos et al., 2014	Burgos et al., 2014
hsa-miR-128-1-5p		Burgos et al., 2014	Burgos et al., 2014
hsa-miR-128-3p		Burgos et al., 2014	Burgos et al., 2014
hsa-miR-1299^∗^	Biofilm formation (*V. cholerae*)	Burgos et al., 2014	Burgos et al., 2014
hsa-miR-132-3p		Burgos et al., 2014	Burgos et al., 2014
hsa-mir-132-5p^∗^, ^∗∗^	Biofilm formation (*E. coli*), Bacterial Secretion Pathway, Quorum Sensing	Burgos et al., 2014; Gui et al., 2015	Burgos et al., 2014; Gui et al., 2015
hsa-miR-134-5p		Burgos et al., 2014	Burgos et al., 2014
hsa-mir-136-3p		Burgos et al., 2014; Gui et al., 2015	Burgos et al., 2014; Gui et al., 2015
hsa-miR-136-5p^∗^	Biofilm formation (*E. coli*), Quorum Sensing	Burgos et al., 2014	Burgos et al., 2014
hsa-miR-138-1-3p		Burgos et al., 2014	Burgos et al., 2014
hsa-miR-138-5p^∗^	Biofilm formation (*E. coli*), Quorum Sensing	Burgos et al., 2014	Burgos et al., 2014
hsa-miR-139-3p		Burgos et al., 2014	Burgos et al., 2014
hsa-miR-139-5p		Burgos et al., 2014	Burgos et al., 2014
hsa-miR-150-5p		Lugli et al., 2015	Burgos et al., 2014
hsa-mir-151		Gui et al., 2015	Gui et al., 2015
hsa-mir-16-2		Gui et al., 2015	Gui et al., 2015
hsa-miR-212-3p		Burgos et al., 2014	Burgos et al., 2014
hsa-mir-29c^∗∗^		Gui et al., 2015	Gui et al., 2015
hsa-miR-323a-3p		Burgos et al., 2014	Burgos et al., 2014
hsa-miR-323b-3p		Burgos et al., 2014	Burgos et al., 2014
hsa-mir-331-5p		Gui et al., 2015	Gui et al., 2015
hsa-miR-370-3p		Burgos et al., 2014	Burgos et al., 2014
hsa-miR-376a-5p		Burgos et al., 2014	Burgos et al., 2014
hsa-miR-377-5p		Burgos et al., 2014	Burgos et al., 2014
hsa-miR-383-5p		Burgos et al., 2014	Burgos et al., 2014
hsa-miR-409-3p		Burgos et al., 2014	Burgos et al., 2014
hsa-miR-431-3p		Burgos et al., 2014	Burgos et al., 2014
hsa-miR-433-3p		Burgos et al., 2014	Burgos et al., 2014
hsa-miR-485-5p		Burgos et al., 2014	Burgos et al., 2014
hsa-miR-539-5p		Burgos et al., 2014	Burgos et al., 2014
hsa-miR-744-3p		Burgos et al., 2014	Burgos et al., 2014
hsa-miR-760		Burgos et al., 2014	Burgos et al., 2014


*^∗^miRNAs that are involved in one of the analyzed pathways (see Results: Pathway analysis); ^∗∗^miRNAs that have been chosen for selective analysis (see Results: Selection of miRNAs)*.

### Crosstalk Between miRNA and Microbiome Composition

**FIGURE 2:**
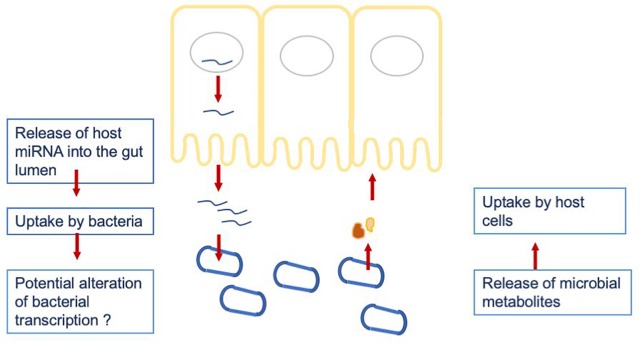
Hypothesized crosstalk between the host miRNAs being released into the gut lumen and the gut microbiome.

To investigate a potential crosstalk between bacteria and host miRNAs, as illustrated in Figure 2, we aimed to detect potential target sites of miRNAs within bacterial genomes. All 346 miRNAs were screened against 207 bacterial reference genomes, taken from a database of human gut metagenomic samples (listed in Supplementary Tables S3, S4). The most prevalent bacterial phyla in the database were Firmicutes, Proteobacteria and Bacteroidetes. The target scan using miRanda (v3.3a; Enright et al., 2003) revealed 20363 potential binding sites on the 205 bacterial reference genomes for 327 unique miRNAs. It is worth mentioning that each miRNA has the potential to target multiple locations and vice versa; a particular region can be associated to multiple miRNAs. Interestingly, eleven out of the selected miRNAs were connected to over 200 protein identifiers. Here, the miRNA hsa-miR-1226-5p had the maximum amount of potential target sites with 1109 hits predicted by miRanda, followed by hsa-miR-1183 with 780, hsa-miR-4767 with 683 and hsa-miR-3916 with 604 possible binding sites.

**FIGURE 3:**
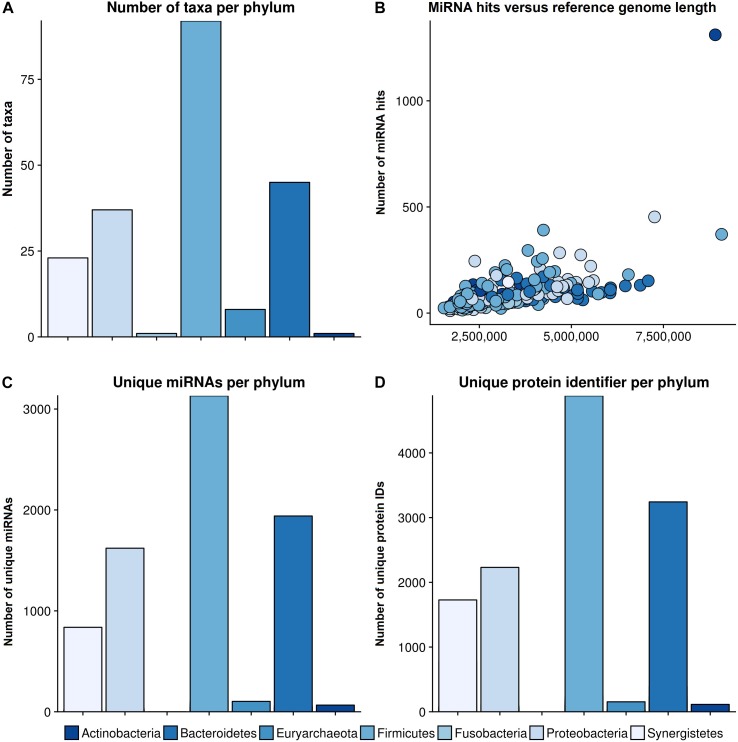
General results of the target scan. **(A)** The species composition of our genomic reference database. **(B)** The number of miRNA hits plotted against the reference genome length of the different phyla. **(C)** The number of miRNAs that revealed hits with a bacterial organism is shown per phylum, and **(D)** the number of unique protein IDs, which were predicted to have putative target sites for miRNAs, in different phyla. The colors refer to different phyla. The legend for all graphs is given beneath the lower plots.

The bar plot in Figure 3A shows the number of different species per phylum of the reference database. The largest phylum with the highest number of different species is Firmicutes, followed by Proteobacteria and Bacteroidetes. The correlation between total miRNA hits, as predicted by miRanda, and the length of the reference genomes per taxonomy identifier is shown in Figure 3B. As expected, the number of hits grows with genome length. The outlier on the top right is *Streptomyces sparsogenes*. Figure 3C shows the miRNA match per phylum for the complete set of miRNAs. Similar to the reference database, Firmicutes, Proteobacteria and Bacteroidetes were the most prominent taxa. In Figure 3D the number of unique proteins targeted in the respective phyla are given. It is evident here that Firmicutes and Proteobacteria have the most hits. Compared to the hits of unique miRNAs, the ratio of unique proteins that were targeted between Firmicutes and Proteobacteria is shifted toward the latter. Moreover, Synergistetes were targeted at a much higher frequency. Upon closer examination, the counts for protein identifiers in Actinobacteria were mostly composed of *Streptomyces sparsogenes* targeted by hsa-miR-4767. This combination accounted for 203 of 683 hits.

### Investigation of Potential Targets by Screening KEGG Pathways

Because several bacterial biofilm components (like functional amyloids or bacterial endotoxins) are linked to the aggravation of symptoms in AD and PD, we screened a number of reference pathways from the Kyoto Encyclopedia of Genes and Genomes (KEGG) Orthology (KO) Database for proteins that were targeted by the constructed miRNA set (Ogata et al., 1999; Kanehisa and Goto, 2000; Kanehisa et al., 2016, 2017). Pathways of particular interest were the bacterial secretion system, lipopolysaccharide biosynthesis and the biofilm formation pathway of three different species.

It is worth mentioning that only protein IDs mapped to a KO pathway could be considered in the analysis.

Furthermore, several species were mapped to different pathways simultaneously.

#### Bacterial Secretion System

The bacterial secretion system is mainly responsible for shuttling proteins across cell membranes. Furthermore, bacterial secretion is linked to virulence and interaction with the host’s immune system. Especially in the case of AD, latent infection is increasingly recognized as a potential cause for the disease (Green and Mecsas, 2016; Fülöp et al., 2018).

**FIGURE 4:**
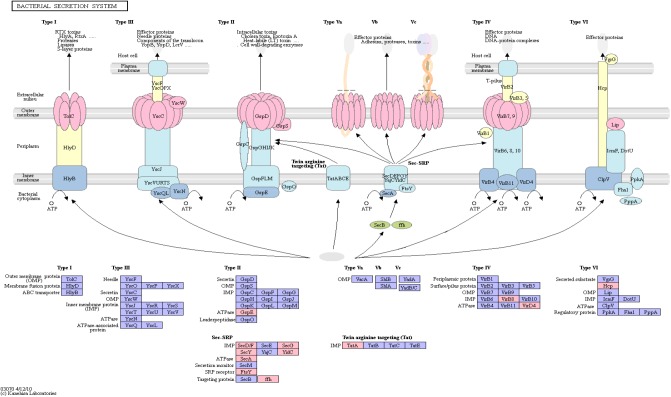
Bacterial Secretion System from KEGG. Proteins encoded by bacterial genome regions that revealed miRNA target matches are colored in pink.

Figure 4 shows an overview of the bacterial secretion system from KEGG. Among the successfully mapped identifiers, mostly Type II secretion pathway proteins were targeted. Those belong primarily to the Sec-SRP class, that is shown in Figure 4. The targeted identifiers (gspE, secA, secF, secG, secY, SRP54, ftsY, tatA, virB8, virD4, yidC) revealed an overlap with the genomic positions of 20 original protein IDs for 16 miRNAs on 19 taxonomic units (Supplementary Table S5). miRNA hsa-miR-3180-5p targeted three different proteins (secA, secF and gspE) in three different species (*Ruminococcus champanellensis* 18P13 = JCM 17042, *Pseudomonas* sp. ADP, *Klebsiella* sp. 4_1_44FAA). hsa-miR-132-5p and hsa-miR-1183 targeted two protein IDs in two different species, respectively.

#### Lipopolysaccharide Biosynthesis

**FIGURE 5:**
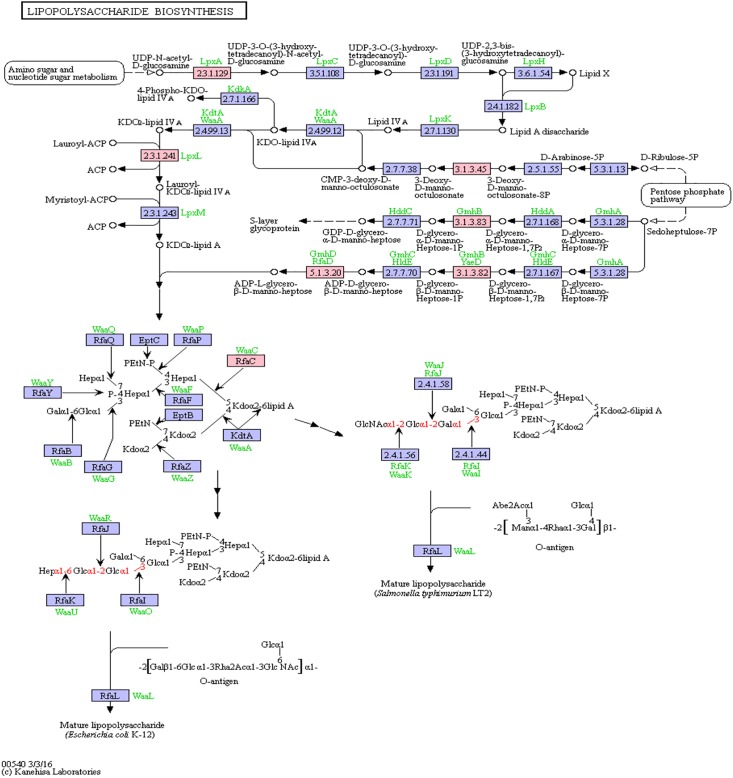
Lipopolysaccharide Biosynthesis from KEGG. Proteins encoded by bacterial genome regions that revealed miRNA target matches are colored in pink.

Bacterial lipopolysaccharides (LPS) are known to trigger a widespread host immune system response as well as neuroinflammation (Poltorak et al., 1998; Li et al., 2016). Increased LPS levels were found in both, the bloodstream and the brain of AD patients (Zhao et al., 2017; Zhan et al., 2018). Assessment of pathway members of the bacterial lipopolysaccharide biosynthesis pathway that harbor putative miRNA binding sites led to six hits, namely: lpxA, lpxL, waaC, kdsC, gmhB, gmhD. In Figure 5, the lipopolysaccharide biosynthesis is shown with protein hits colored in pink.

From the list of targeting miRNAs, those six proteins had 7 hits on 6 taxonomic entries, belonging to the Bacteroidetes and the Proteobacteria. Among all hits, only hsa-miR-3180-5p targeted the same protein in two different *Klebsiella* species, namely lpxL (Supplementary Table S5).

#### Biofilm Formation

**FIGURE 6:**
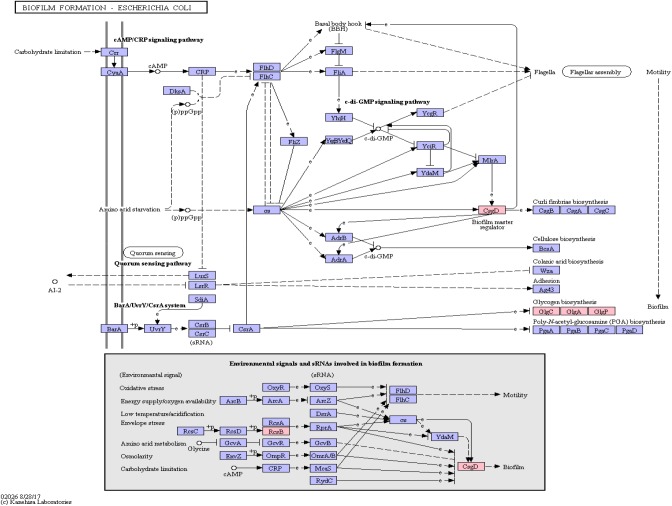
Biofilm Formation Pathway using KEGG pathway mapping for *E. coli*. Proteins encoded by bacterial genome regions that revealed miRNA target matches are colored in pink.

Functional amyloids, such as curli, are often components of bacterial biofilms (Sawyer et al., 2012) which have the potential to change the virulence and invasiveness of bacteria. Furthermore, it has been shown that biofilm components were able to increase amyloidogenesis, even when only fed to rats (Chen et al., 2016). We examined this pathway class via KEGG pathway mapping for the organism *Escherichia coli* (see Table 3 and Figure 6) as well as for *Vibrio cholerae* (Supplementary Figure S1) and *Pseudomonas aeruginosa* (Supplementary Figure S2).

**Table 3:** List of mapping proteins in biofilm formation of *Vibrio cholerae, Escherichia coli*, and *Pseudomonas aeruginosa*.

*Vibrio cholerae*	*Escherichia coli*	*Pseudomonas aeruginosa*
CysE	PYG	ImpH
WecB	GlgA	Hcp
PaaK	GlgC	
GspE	CsgD	
WecC	RcsB	
RpoN		
Fis		


Interestingly, CsgD, a master regulator of biofilm formation and the synthesis of curli fimbriae (Brombacher et al., 2006), was targeted on *Klebsiella* sp. 4_1_44FAA. Since this is a multispecies protein for Enterobacteriaceae, it also maps in the *E. coli* pathway map. Other proteins targeted are PYG, GlgA and GlgC, as shown in Figure 6. All these proteins are involved in the glycogen biosynthesis. For the *Vibrio cholerae* pathway map (Supplementary Figure S1), seven proteins offered target sites for miRNAs in the target scan. WecC is involved in the biosynthesis of polysaccharides. RpoN seems to be involved in bacterial virulence (Kazmierczak et al., 2005). Fis is involved in flagellar movement and controls virulence-associated genes (Jakovleva et al., 2012). GspE is a protein involved in the general secretion pathway. PaaK is associated with phenylactetat-CoA ligase, an enzyme involved in energy metabolism. WecB seems to be involved in capsule precursor proteins (Swartley et al., 1998). miRNA targets within *Pseudomonas aeruginosa* were ImpH and Hcp (for pathway overview see Supplementary Figure S2). ImpH is involved in transport through membranes, while Hcp seems to be involved in the interaction of *E. coli* with human endothelial cells (Zhou et al., 2012).

### Target Scan Results for a Subset of miRNAs

**FIGURE 7:**
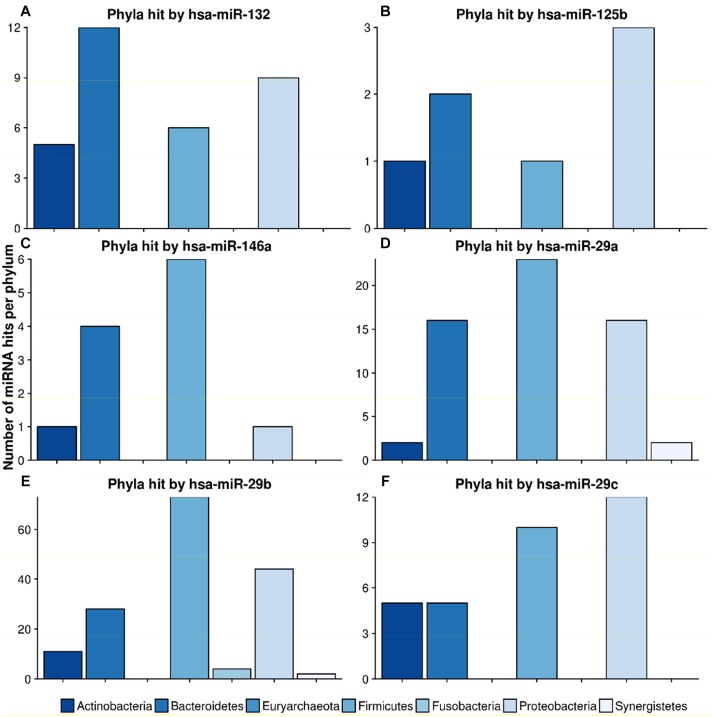
Number of hits from specific miRNAs per phylum. The bar plots show the number of hits from miRNAs **(A)** hsa-miR-132, **(B)** hsa-miR-125b, **(C)** hsa-miR-146a, **(D)** hsa-miR-29a, **(E)** hsa-miR-29b and **(F)** hsa-miR-29c counted for different phyla. The legend can be found at the bottom. The colors refer to different phyla.

To investigate the impact of individual miRNAs on potential bacterial targets, we shortlisted six miRNAs based on systematic literature research, as well as those two miRNAs who had the highest amount of hits, according to the target scan.

mir-132, a well-known mediator of neurodegenerative diseases (Hernandez-Rapp et al., 2017; El Fatimy et al., 2018), targeted 32 different species in 4 different phyla. Figure 7 shows the miRNA hits on annotated proteins per phylum. It becomes evident that, compared to the other phyla, Bacteroidetes are more frequently targeted, despite the fact that they are only the third largest phylum in the overall taxonomic composition of the database. Conversely, Firmicutes, representing the largest taxonomic group in the database were targeted at a much lower frequency.

The scan for the mir-29 family revealed that they all mostly target Firmicutes, Proteobacteria and Bacteroidetes at the same ratio. Mir-29b, with 162 species in 6 phyla, had the highest number of predicted target sites. miR-125b had most hits on the phylum Proteobacteria, followed by Bacteroidetes. Mir-146, a miRNA associated with the immune system response and upregulation in AD (Arroyo et al., 2018), targeted mainly Firmicutes and Bacteroidetes, with only one hit on Proteobacteria and Actinobacteria, respectively.

A recent study by Du et al. (2018) showed that overexpression of miR-146a is linked to an altered gut microbiome in mice. Supplementary Table S7 lists the exact hits for any protein and any species that could potentially be targeted by hsa-miR-146a-5p. With this in mind, we investigated the taxa that were downregulated upon mir-146a upregulation. Out of the 45 Proteobacteria present in our database, hsa-miR-146a-5p targeted exactly 1 species, *Desulfovibrio* sp. A2, on one protein, the chromosomal replication inhibitor protein *DnaA*. Within the seven Prevotellaceae, *Prevotella salivae* DSM was targeted on the diguanylate cyclase. Parasutella was not targeted at all, since this species was not contained in our gut database. According to Du et al. (2018), overabundant taxa were Alistipes, Blautia, Coprococcus and Ruminococcus. For this list of taxa, no targets were predicted for hsa-miR-146a-5p. In conclusion, we found two potential hits on taxa that were downregulated and none on taxa that were upregulated in Du et al. (2018) given our genomic database.

**FIGURE 8:**
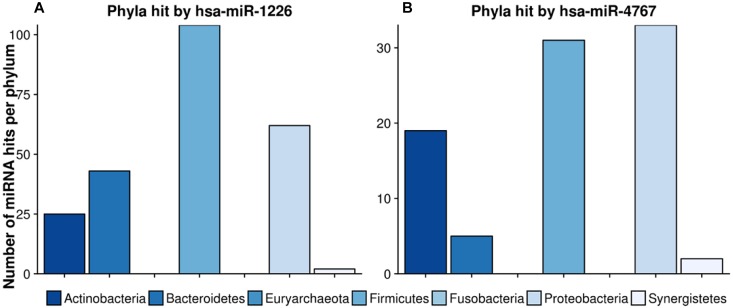
Number of hits per phylum from miRNAs with the largest target matching. In **(A)**, the hit counts of hsa-miR-1226 per phylum are given, in **(B)** the number of targeted regions of hsa-miR-4767 counted for different phyla. The legend can be found at the bottom. The colors refer to different phyla.

Figure 8 highlights two very interesting miRNAs, namely hsa-miR-1226 and hsa-miR-4767, which were selected due to their high number of potential target sites reported by miRanda (see section “Crosstalk Between miRNA and Microbiome Composition”).

hsa-miR-1226 was predicted to have in total 1109 matches whereas hsa-miR-4767 had 683 hits. Interestingly, the latter had an unexpected high amount of 203 hits on *Streptomyces sparsogenes*, accounting for almost one third of the total amount of hits. In both cases, highly targeted phyla were Firmicutes as well as Proteobacteria. Supplementary Table S6 lists the GO-terms for the selected miRNAs.

Finally, we were interested in putative targets hits on serine proteases. Bacterial HTRA serine proteases have been implicated to play a role in the cleavage of the amyloid precursor protein. Their inhibition causes β-amyloid to accumulate in a culture of astrocyte cells (Grau et al., 2005). A first examination revealed two hits on serine proteases in *Lactobacillus antri* and *Flavonifractor plautii* by hsa-miR-664a-5p and hsa-miR-1248, respectively.

## Discussion

The search for a cure for neurodegenerative diseases related to protein misfolding such as AD and PD has also led to studies of the gut microbiome and its potential dysregulation. Different studies have demonstrated a connection between the etiology of AD or PD and microbiome alterations (Cattaneo et al., 2017; Vogt et al., 2017). However, the question still remains if and how exactly the host communicates with its internal gut microbiota. A novel and interesting link between host and bacteria was demonstrated by Liu et al. (2016), who showed that miRNAs from the host might be able to enter bacterial cells and downregulate the expression of bacterial target sequences (Liu et al., 2016). Additionally, sequences as short as miRNAs and acting like miRNAs have been naturally found in bacteria (Nejman-Faleńczyk et al., 2015), giving further evidence that small RNAs of this size might be active in bacteria. We therefore hypothesized the existence of a crosstalk between microRNAs commonly associated with AD or PD in humans and target sites on bacterial genomes within the human gut metagenome.

Another potential fit for this size requirement are external guide sequences that mediate cleavage by RNAse P (Davies-Sala et al., 2015). These sequences consist of about 13 to 19 nucleotides complementary to their target site and possess a 3’RCCA nucleotide signature necessary for the cleavage. The only miRNA showing hits and meeting these criteria was hsa-miR-770-5p.

We screened a broad initial dataset of over 300 miRNAs in order to gain a detailed overview over potential interactions between miRNAs and the gut metagenome. The first target scan on gut bacterial genomes for miRNAs involved in AD and PD revealed hits for almost all of the microRNAs investigated in this study, resulting in a plethora of potential interactions.

The sheer volume of interactions predicted by miRanda made it necessary to further reduce the set of targets. This was accomplished by focusing on all hits on bacterial pathways that were assumed relevant in AD and/or PD as well as a core set of miRNAs prominently highlighted in previous studies for their involvement in the microbiome in AD/PD.

The initial results of the target screen made evident that some miRNAs target multiple proteins. This went as far as 1109 predicted hits on protein IDs for hsa-miR-1226-5p. While some of these hits may certainly be false positives, miR-1226-5p was among the miRNAs investigated in Liu et al. (2016), where it upregulated the growth of *E. coli*. In the same vein, the miRNAs hsa-miR-1183, hsa-miR-4767, hsa-miR-3916, hsa-miR-3180-5p, hsa-miR-1248, hsa-miR-1301-3p, hsa-miR-378c, hsa-miR-1538, hsa-miR-671-5p and hsa-miR-939-5p show promise for future investigations. Each one had over 200 hits of protein identifiers. These could either be spurious hits on repetitive sequences, devoid of any functional meaning, or hits on conserved protein stretches, enabling a single miRNA to target a functional class of proteins over a broad array of species. For eukaryotes it is known that particular miRNA can target several proteins connected in one pathway and that some miRNA families are highly conserved (Kehl et al., 2017).

Since we performed only an *in silico* scan for miRNA hits on bacterial reference genomes, any detected target requires validation. At the present time, the necessary circumstances that have to be met for a human miRNA to be effective in bacteria are unclear. Properties like target seed site conservation are well known features in eukaryotic miRNAs. If and how this feature might be present in miRNA hits on bacterial sequences, remains to be seen (Ellwanger et al., 2011).

Moreover, data regarding fecal miRNA are lacking for AD and PD compared to data for blood and other biofluids and tissues. While it is possible to separate the miRNAs into groups of up- or down-regulated, this might be misleading for several reasons. Due to the lack of data, we did not investigate a single study containing fecal miRNA in AD or PD. Therefore, we do not know if the composition differs between body sites. Even within the same body fluids, different studies showed discordant results. Since it is unclear how miRNAs are exactly imported into and processed within bacteria, selective import might change the ratios of the relevant miRNAs.

As the next step, bacterial pathways and protein cascades with a known involvement in AD or PD were searched. It needs to be mentioned at this point that we only considered the identifiers that could be mapped to KEGG Pathways, as well as some manual exceptions. This includes the lipopolysaccharide biosynthesis. Lipopolysaccharides (LPS) of *E. coli* are known to be elevated in the brain of patients with AD. Furthermore, it has been hypothesized that gut derived LPS are able to traverse the intestinal barrier and travel to the brain. LPS derived from gram negative bacteria colocalises with plaques. Additionally, LPS levels are elevated in the bloodstream of AD patients. This pattern fits well with other studies attesting a link between neuroinflammation and AD (Zhao et al., 2017; Zhan et al., 2018). In PD, abnormal levels of an LPS-binding protein were found to be a biomarker for both gastrointestinal inflammation and PD (Pal et al., 2015).

The next pathway we evaluated was biofilm formation. Biofilm formation leads to a multitude of functional adaptations for the bacteria. Bacteria are able to secrete numerous functional amyloids, among them Curli, Fap, HpaG and FUBA. With respect to the etiology of AD and PD, it is known that bacterial amyloids are able to propagate amyloid formation *in vivo*. This is supported by rodent studies, where rats that were fed with bacterial amyloids showed enhanced alpha synuclein formation in the brain. This leads to the speculation that biofilm components from the gut microbiota are able to cross-seed to the brain, where they induce amyloidogenesis (Lundmark et al., 2005; Chen et al., 2016; Del Tredici and Braak, 2016). More indirect links between biofilm formation and pathogenesis are connected to increased cell adhesion, increased virulence contributing to “leaky gut” and a general weakening of endothelial barriers, observed as well in AD as in PD (Sawyer et al., 2012; Friedland and Chapman, 2017).

Secretion across lipid membranes is linked to virulence and host immune evasion (Green and Mecsas, 2016). Especially with respect to AD, there is a paradigm shift toward latent chronic infection as a main cause for the disease (Fülöp et al., 2018). Coincidentally, one of the first validated functions for a micro-RNA-sized RNA in bacteria was regulating the virulence of said bacteria (Nejman-Faleńczyk et al., 2015).

Microbiome dysbiosis in general has been previously recognized in both, AD and PD, and there are several studies, that found different abundances of bacteria between cases and controls through the analysis of fecal 16S rRNA (Vogt et al., 2017; Qian et al., 2018; Zhuang et al., 2018). Some of the species mentioned here were regrettably not present in our genomic database, but, for example, Ruminococcaceae had target site predictions on all of their 12 members in our collection of bacterial genomes. That differential expression of a miRNA, in principle, can alter bacterial abundance has been shown by Du et al. (2018), who knocked out miR-146 in mice. If the miRNA-microbiome crosstalk should prove true, it would be definitively worth to investigate further whether the targeting miRNAs have an influence on the species composition of the microbiome by regulating bacterial transcription.

For future studies an *in vivo* validation of putative hits is necessary. Furthermore, since most known bacterial small RNAs are >50–200 nucleotides (Choi et al., 2017), considering longer transcripts might provide further insights. In addition, other classes of small RNAs such as tRNA fragments, which are known to be associated to regulatory pathways in other species, could be considered.

Conclusively, our *in silico* target screen found several miRNAs that may represent key regulators in bacterial pathways relevant to AD or PD. If the human miRNAs should prove to be effective in regulating bacterial transcription, this could lead to several new insights regarding both the microbiome host interaction and the treatment of neurodegenerative diseases. As such, the microbiome-miRNA crosstalk might contribute to the idea of what constitutes a “healthy microbiome,” beyond the mere abundance of bacterial taxa toward differential regulations of global pathways. Moreover, there are currently several miRNAs undergoing clinical studies for their use as therapeutics for example against cancer or hepatitis (Rupaimoole and Slack, 2017). Thus, the miRNAs might be used as a therapy option to revert the microbiome to a desired state. This may even be applied years before the disease breaks out, so as to keep the pathological changes beyond a threshold level in order to prevent the outbreak of the disease altogether.

## Data Availability

Publicly available datasets were analyzed in this study. This data can be found here:

https://exrna-atlas.org/exat/publicAnalysis/DESeq2/KJENS1_ADPD_PD_VS_CONTROLS_CSF

https://exrna-atlas.org/exat/publicAnalysis/DESeq2/KJENS1_ADPD_AD_VS_CONTROLS_SERUM

https://exrna-atlas.org/exat/publicAnalysis/DESeq2/KJENS1_ADPD_AD_VS_CONTROLS_CSF

https://exrna-atlas.org/exat/publicAnalysis/DESeq2/KJENS1_ADPD_AD_VS_PD_SERUM

https://exrna-atlas.org/exat/publicAnalysis/DESeq2/KJENS1_ADPD_AD_VS_PD_CSF

https://exrna-atlas.org/exat/publicAnalysis/DESeq2/KJENS1_ADPD_PD_VS_CONTROLS_SERUM

ERCC: Ainsztein et al. (2015) – Original data can be found under phs000727.v1.p1 in dbgap.

## Author Contributions

CH wrote the manuscript, performed the analysis, and interpreted and visualized the results. JK, AW and JL contributed to writing the manuscript, performed parts of the analysis, and visualized the results. CR edited the manuscript and interpreted the results. KE and SG initiated and supervised the study, interpreted the results, and edited the manuscript. All authors read and approved the final manuscript.

## Conflict of Interest Statement

The authors declare that the research was conducted in the absence of any commercial or financial relationships that could be construed as a potential conflict of interest.

## Supplementary Material

FIGURE S1 Biofilm formation pathway of Vibrio cholerae from KEGG. Protein IDs that were associated to miRNAs are colored in pink.

FIGURE S2 Biofilm formation pathway of *Pseudomonas aeruginosa* from KEGG. Protein IDs that were associated to miRNAs are colored in pink.

TABLE S1 List of all studies that were used in this study; including miRNA and microbiome studies in relation to Alzheimer’s and/or Parkinson’s disease.

TABLE S2 List of differentially expressed miRNA between AD and Control, PD and Control and AD and PD; from literature including 7 human studies (see Supplementary Table S1).

TABLE S3 List of all 346 miRNA IDs that have been used for target analysis (see Supplementary Tables S1, S2).

TABLE S4 List of all 207 bacterial genome accession IDs, including species name and taxonomy ID.

TABLE S5 Results of the pathway analysis. Each table refers to one pathway and lists the identified protein ID, its associated taxonomy ID, miRNA ID and expression in AD and/or PD from literature.

TABLE S6 Plots of the most interesting miRNAs and the GO-terms that they are targeting. The additional tables help to see which function the GO-term has.

TABLE S7 Exact hits for any protein and any species that could potentially be targeted by hsa-miR-146a-5p.

TABLE S8 Metadata for patients in the seven miRNA studies related to AD or PD.
