# Supplementary figures and images for: Common miRNA Patterns of Alzheimer’s Disease and Parkinson’s Disease and Their Putative Impact on Commensal Gut Microbiota
Source: Front Neurosci. 2019 Mar 5;13:113. doi: 10.3389/fnins.2019.00113 (PMC6411762; doi:10.3389/fnins.2019.00113)

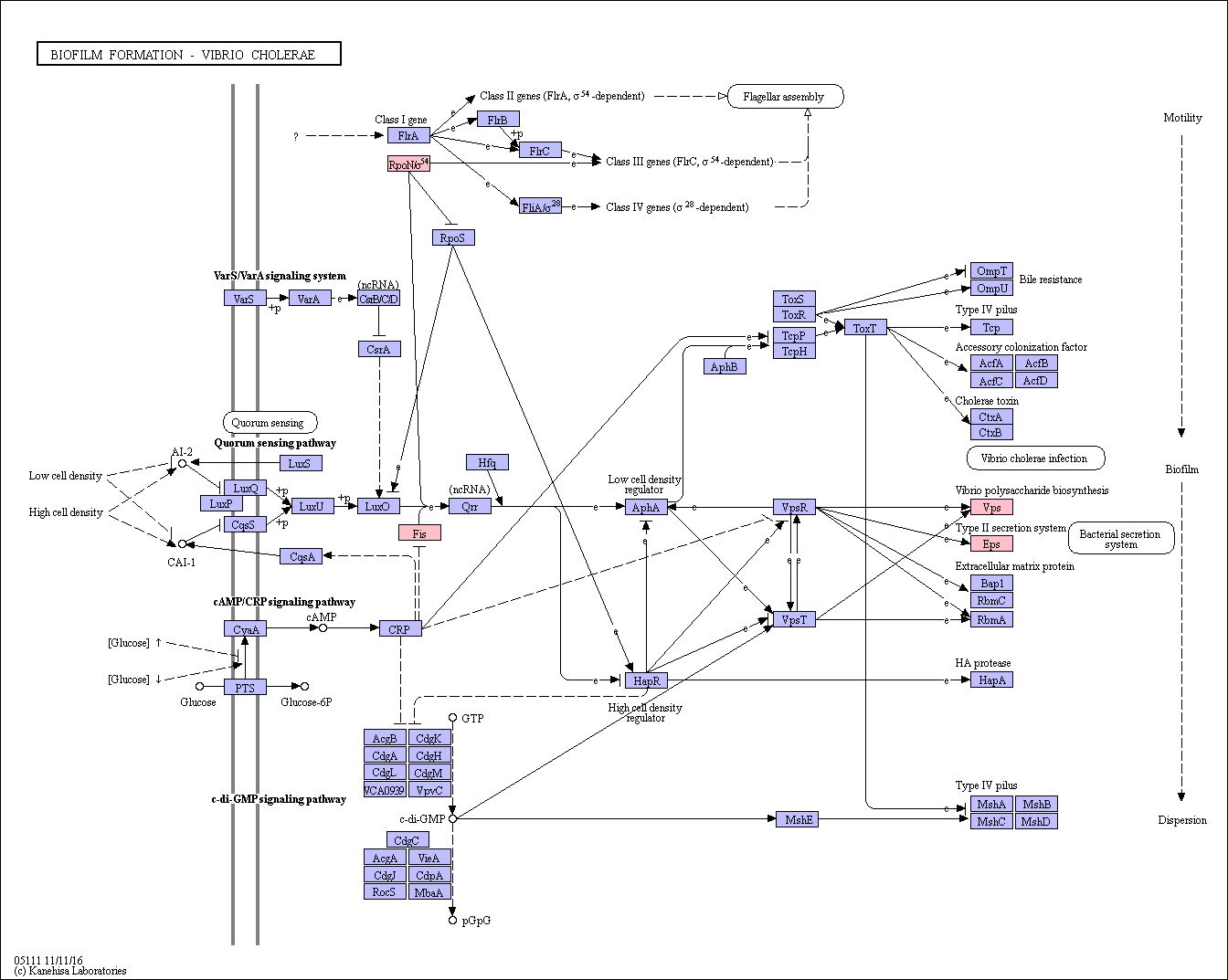

Supplement: FIGURE S1 — Biofilm formation pathway of Vibrio cholerae from KEGG. Protein IDs that were associated to miRNAs are colored in pink. [file Image_1.jpeg]

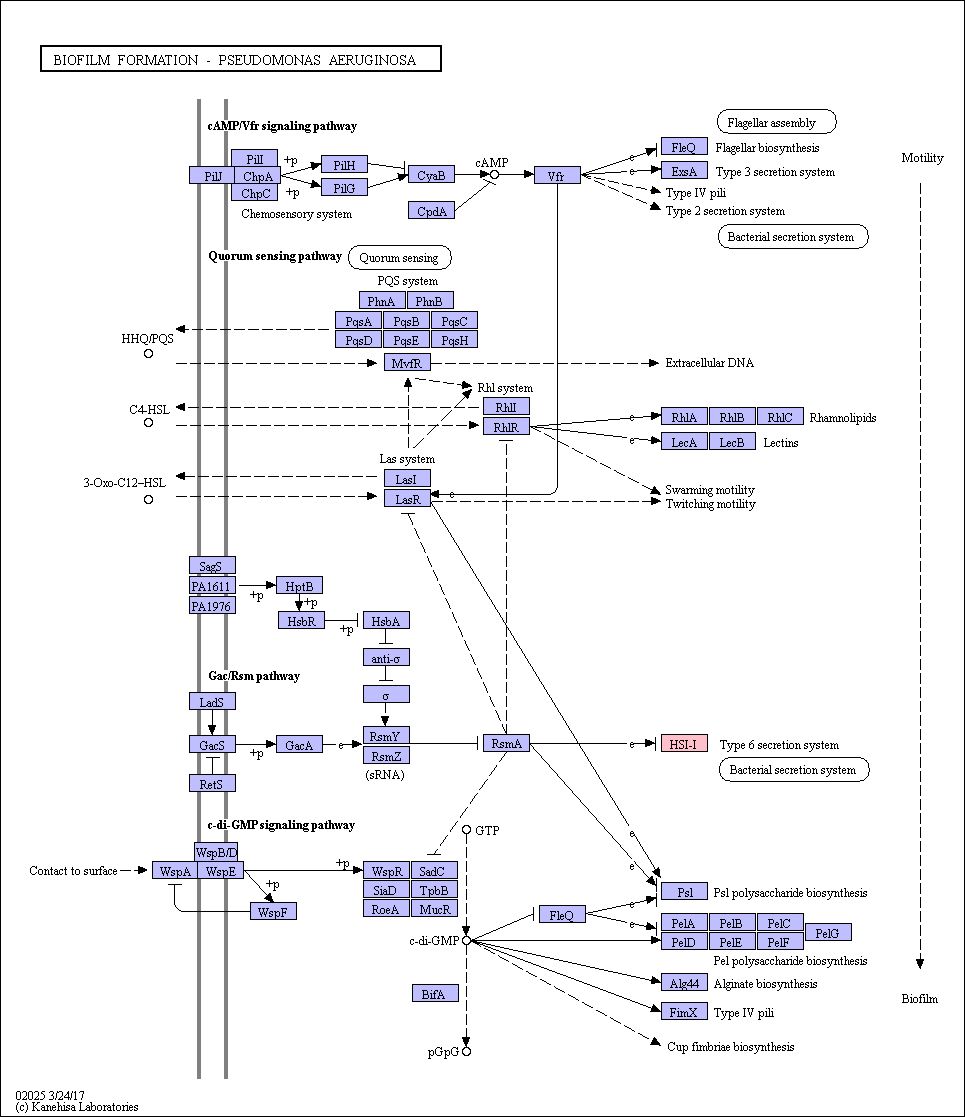

Supplement: FIGURE S2 — Biofilm formation pathway of Pseudomonas aeruginosa from KEGG. Protein IDs that were associated to miRNAs are colored in pink. [file Image_2.jpeg]
