# Supplementary material for: Common miRNA Patterns of Alzheimer’s Disease and Parkinson’s Disease and Their Putative Impact on Commensal Gut Microbiota
Source: Front Neurosci. 2019 Mar 5;13:113. doi: 10.3389/fnins.2019.00113 (PMC6411762; doi:10.3389/fnins.2019.00113)
Supplement: TABLE S8 — Metadata for patients in the seven miRNA studies related to AD or PD. [file Table_8.docx]

**Metadata for patients in the 7 miRNA studies related to AD or PD**

(Aggregated from the M&M sections and the Supplements of the original publications, links to the main publications can be found in Table S2)

**study 1 Burgos et al., 2014**

Patients :&

Controls: “were enrolled in the Banner Sun Health Research Institute (BSHRI) Brain and Body Donation Program as a whole-body donor.”

No variation in “samples due to age, gender, or postmortem interval”.

Drugs: No mention if anti inflammatory drugs were used.

Demography: No explicit mention of demography.

Age C: ca 82 yrs, PD ca 80 yrs, AD ca 81 yrs (mean values)

Gender: overall 118 M + 98 F.

**study 2 Martins et al., 2011**

Patients: 19, had more than 2 years of PD (classif: Hoehr & Yang and Schwab and England and UPDRS)

Controls: 13, had no PD and no family history of PD

Age: PD: ca 65 yrs; C: ca 60 yrs

Gender C: 5M/8F; PD: 10M/9F

Demography: Portuguese Caucasians

Drugs: No explicit mention of anti inflammatory drugs.

**study 3 Soreq et al., 2013**

Patients: 6, (classif. UPDRS-III)

Controls: 7, Controls were recruited among Hadassah hospital staff

and researchers at the Edmond J. Safra Campus (Jerusalem)

Gender: all male

Age: age matched

Exclusion of the conditions:

depression and past and current DSM Axis I and II psychological disorders (SM), chronic inflammatory disease, coagulation irregularities, previous malignancies or cardiac events, or any surgical procedure up to 1 year pre-DBS.

However included: hyperlipidemia(two patients and three controls), hypertension and diabetes (two patients each).

Drugs: Two patients received anti-hypertension medication and one-hyperlypidaemia treatment, dopamine replacement therapy

Demography: No explicit mention of demography.

**study 4 Lugli et al., 2015**

Patients: “Clinic participants presented to the Rush Memory Clinic, a specialty referral clinic at the Rush University Medical Center, for evaluation of their cognition, as previously described [30, 31]. Clinic participants underwent uniform, structured, clinical evaluations. As previously reported [32], evaluations included a detailed medical history, neurologic examination, cognitive function testing, brief psychiatric evaluation, and an interview with a knowledgeable informant. Ancillary tests (e.g., laboratory testing, structural neuroimaging, examination of cerebrospinal fluid, positron emission tomography) were obtained when clinically indicated. The procedures were compatible with the Consortium to Establish a Registry for Alzheimer’s Disease (CERAD) [33], similar to those conducted by the Clinical Cores of other federally-funded AD Centers, and consistent with the current practice parameters for the diagnostic evaluation for dementia [34].”

Controls: “Community participants mainly were spouses of persons diagnosed with dementia due to Alzheimer’s disease. As part of the Rush Memory Clinic Data Repository, the community participants without dementia (n = 41) had a recorded medical history which included self-report about not being diagnosed with dementia. The majority (n = 32) also had screening cognitive testing obtained in person or via the telephone. Community participants diagnosed with dementia due to Alzheimer’s disease (n = 4) had medical records requested for review regarding the diagnosis”

Drugs : Exclusion of persons on heparin therapy.

Age: 50 and 75

Gender: mixed

Demography: No explicit mention of demography

**study 5 Ding et al., 2016**

Patients: 15 (classif: UPDRS-III scores and a modified Hoehn–Yahr scale )

Controls: 15

Exclusion of patients with “cancer, significant cardiac dysfunction or diabetes”.

Controls were deemed “showing no evidence of disease”.

Age: about 60 for both groups

Gender: 2/3 male, 1/3 female

Drugs: No drugs were explicitly mentioned

Demography: Demography was not explicitly mentioned

**study 6 Tatura et al., 2016**

Patients: 22

Controls: 10

Gender: Patients 54% female/46% male; Controls 60% female/40% male

Drugs: No explicit mention of anti inflammatory drugs.

Age: Patients 73.9 years ± 6.9 ; Controls 65.7 ± 10.9 years;

Demography: Caucasian

**study 7 Gui et al., 2015**

Patients: AD: 28 PD: 47

Controls: 27

Gender: C: 8F/9M, AD 13F/15M, PD 22F/25M

Drugs: No explicit mention of anti inflammatory drugs.

Age: C: ca 60, AD ca 65 , PD ca 63 yrs

Demography: No explicit mention
